# Heparin-Coated Tunneled Hemodialysis Catheters Improve Failure-Free Survival in ESRD

**DOI:** 10.3390/medicina62050804

**Published:** 2026-04-22

**Authors:** Volkan Tasci, Ali Fuat Tekin, Yunus Emre Tanrıkulu, İsmail Özer, Onur Taydas, Mustafa Ozdemir, Omer Faruk Topaloglu, Mahmud Islam, Mehmet Halil Öztürk

**Affiliations:** 1 Department of Radiology, Division of Interventional Radiology, Faculty of Medicine, Sakarya University, Sakarya 54050, Türkiye; md.volkantasci@gmail.com (V.T.); yetanrikulu@gmail.com (Y.E.T.); ozer815@gmail.com (İ.Ö.); drmstfrd@gmail.com (M.O.); ozturkmh@gmail.com (M.H.Ö.); 2 Department of Radiology and Radiological Science, Johns Hopkins University School of Medicine, Baltimore, MD 21205, USA; aftrad333@gmail.com; 3 Department of Internal Medicine, Division of Nephrology, Faculty of Medicine, Sakarya University, Sakarya 54050, Türkiye; drisleem@gmail.com

**Keywords:** hemodialysis, tunneled catheter, heparin-coated, vascular access, end-stage renal disease, catheter complications, Cox regression, failure-free survival

## Abstract

*Background and Objectives:* Tunneled hemodialysis catheters (TDCs) are essential for vascular access in patients with end-stage renal disease (ESRD). This study retrospectively evaluated the clinical outcomes of permanent TDCs placed at a single center, comparing heparin-coated versus non-heparin-coated catheters. *Materials and Methods:* A total of 189 patients who underwent permanent TDC placement between January 2021 and January 2022 were included. Patients were categorized by catheter type (heparin-coated, *n* = 80; non-heparin-coated, *n* = 109). Catheter failure-free survival was analyzed using the Kaplan–Meier method, with arteriovenous fistula creation treated strictly as a censoring event. Multivariable Cox proportional hazards regression was used to identify independent predictors of catheter failure. *Results:* Complications were significantly more frequent in the non-heparin-coated group (*p* = 0.004). Catheter exchange was required exclusively in the non-heparin-coated group (*p* < 0.001). Kaplan–Meier analysis demonstrated significantly longer failure-free catheter survival in the heparin-coated group (restricted mean 49.0 vs. 41.7 months; Log-Rank *p* < 0.001). On multivariable Cox regression, the complete absence of events in the heparin-coated group yielded a strong protective point estimate (HR = 0.087, 95% CI: 0.004–1.710), rendering individual patient covariates such as INR and age non-significant. *Conclusions:* Heparin-coated TDCs were associated with significantly longer failure-free survival and lower complication rates compared with non-heparin-coated catheters. Due to the low overall event rate, individual patient-level covariates including INR did not reach statistical significance in the multivariable model.

## 1. Introduction

The global prevalence of chronic kidney disease (CKD) and end-stage renal disease (ESRD) continues to rise, placing increasing demands on renal replacement therapies, including hemodialysis [1]. Adequate and reliable vascular access is a cornerstone of successful hemodialysis, and its quality directly influences patient outcomes, hospitalization rates, and quality of life [2]. While arteriovenous (AV) fistulas remain the preferred form of long-term vascular access owing to their superior patency and lower infection rates, a substantial proportion of patients are unable to establish functioning AV fistulas due to inadequate vasculature, repeated access failures, or clinical urgency [2,3]. In these circumstances, tunneled dialysis catheters (TDCs) serve as an indispensable bridge or long-term access option. Indeed, recent long-term institutional experiences demonstrate that TDCs frequently serve not just as temporary bridges, but as extended or even permanent vascular access solutions for a significant subset of these patients [4].

Despite their widespread use, TDCs carry significant risks, including catheter-related bloodstream infection (CRBSI), fibrin sheath formation, catheter thrombosis, and mechanical complications [5]. These complications not only adversely affect dialysis adequacy but are also associated with substantial morbidity and mortality in an already vulnerable patient population [2]. The main challenge in long-term tunneled hemodialysis access is the initial interaction between the blood vessel and the foreign body of the catheter. When the catheter is inserted, plasma proteins, primarily fibrinogen and albumin quickly adsorb onto its surface. This forms a conditioning film that encourages platelet adhesion, leading to the development of a fibrin sheath. Heparin-coated surfaces are designed to disrupt this process. By using covalently bonded heparin, the catheter surface mimics the antithrombotic properties of the natural vascular endothelium. This modification enhances the local inhibition of the coagulation cascade by increasing the activity of antithrombin III, which helps prevent the conversion of fibrinogen to fibrin at the catheter interface. Reducing the initial biofilm formation is expected to not only prolong the functional life of the catheter but also decrease the risk of catheter-related bloodstream infections (CRBSI) by removing the protein-rich substrate needed for bacterial colonization [6]. Critically, this mechanism is distinct from the practice of heparin locking, in which heparin solution is instilled into catheter lumens between dialysis sessions to prevent intraluminal thrombosis. Unlike locking solutions, surface-bound heparin provides continuous passive protection across the outer catheter surface and operates independently of nursing protocol adherence or lock dwell time, representing a device-level rather than practice-level intervention [7].

Several studies have reported favorable outcomes with heparin-coated catheters, although results have been heterogeneous across patient populations and catheter designs [6,7,8]. Furthermore, few studies have applied penalized regression methods to address the statistical challenge of complete separation that arises when one treatment arm experiences zero events, a scenario encountered in the present cohort. The current study addresses these methodological gaps by applying Kaplan–Meier survival analysis with rigorous censoring rules and Firth’s penalized partial likelihood Cox regression, providing a more robust and reproducible estimate of the survival advantage associated with heparin-coated catheters.

In addition to catheter type, patient-related factors such as diabetes mellitus, hypertension, anemia, female sex, and older age have been commonly cited in ESRD populations as potentially influencing vascular fragility and coagulability [5,9]. There is currently no consensus on whether device-level thromboprotection outweighs patient-level risk factors. The present study was conducted to retrospectively evaluate the clinical outcomes of permanent TDCs placed at our institution over a one-year period. Our primary aim was to compare complication rates and failure-free catheter survival between heparin-coated and non-heparin-coated catheters. As a secondary aim, we evaluated potential independent predictors of catheter failure using multivariable survival analysis.

## 2. Materials and Methods

### 2.1. Study Design and Patient Selection

**Figure 1:**
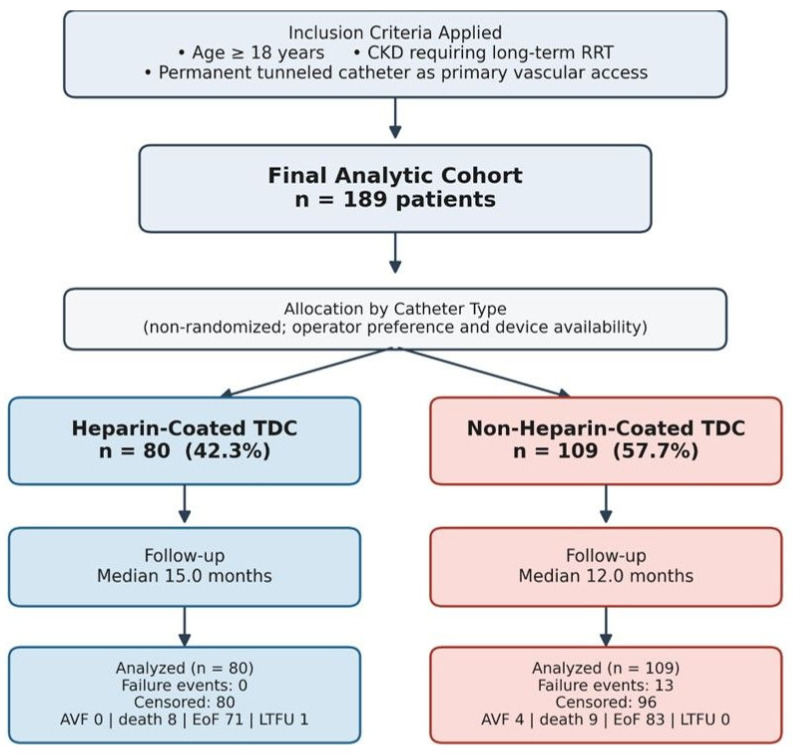
Study design and patient enrollment flowchart. Note: CKD = Chronic kidney disease, RRT = renal replacement therapy, TDC = Tunneled dialysis catheter, AVF = Arteriovenous fistula, EoF = End of follow-up, LTFU = Loss to follow-up.

This single-center, retrospective observational study was conducted in compliance with the principles of the Declaration of Helsinki. Patients who underwent permanent tunneled dialysis catheter placement at our institution between January 2021 and January 2022 were identified from prospectively maintained procedural records. The study followed the Strengthening the Reporting of Observational Studies in Epidemiology (STROBE) guidelines. Inclusion criteria were age ≥ 18 years, chronic kidney disease requiring long-term renal replacement therapy, and permanent tunneled catheter placement as the primary vascular access modality (Figure 1). Patients with incomplete clinical records or those lost to follow-up within 30 days of placement were excluded. The study was approved by the Clinical Research Ethics Committee of Sakarya University Faculty of Medicine (Protocol No.: E.533681; Approval Date: 18 November 2025).

### 2.2. Catheter Placement and Types

All catheter placements were performed by experienced interventional radiologists under real-time ultrasound guidance for venous access and fluoroscopic guidance for catheter positioning. Patients were positioned supine under standard sterile conditions. Continuous monitoring of heart rate, blood pressure, and oxygen saturation was performed. Local anesthesia was administered using prilocaine (Priloc, Vem, Sarıyer, Türkiye) at the puncture and tunneling sites. Prophylactic antibiotics were not routinely administered. Venous access was obtained under real-time ultrasound guidance using a high-frequency linear transducer (7–12 MHz). Following venous puncture, a guidewire was advanced under fluoroscopic guidance. Fluoroscopy was performed using a digital subtraction angiography system with standard frame rates (typically 3–7.5 frames per second). A subcutaneous tunnel was created from the exit site on the anterior chest wall to the venous entry point. The tunnel length was determined to minimize infection risk and ensure patient comfort. The catheter was advanced over the guidewire, and its tip was positioned at the cavoatrial junction or within the right atrium, as confirmed fluoroscopically. Catheter lumens were flushed with saline and locked with heparin solution according to institutional protocol. Catheter function was verified by aspiration and saline infusion. A sterile dressing was applied, and patients were monitored for immediate complications. The right internal jugular vein was the preferred insertion site; the left internal jugular vein and femoral veins were used when the right side was inaccessible. All catheters utilized in this study were 14.5 French in diameter and 19, 23, 28, and 33 cm in length (Medtronic Covidien Palindrome, Mansfield, OH, USA), depending on the patient’s anatomical requirements determined during fluoroscopy. The heparin-coating technology employed in the study group is specifically engineered to maintain its antithrombotic properties throughout the life of the catheter without systemic anticoagulation effects. Catheter type selection was not randomized; it was determined by the treating interventional radiologist based on institutional device availability and operator preference during routine clinical practice. No protocolized patient-specific allocation criteria were applied, and unmeasured confounding related to operator practice patterns or temporal device availability cannot be completely excluded.

### 2.3. Clinical Definitions and Endpoint Criteria

The primary endpoint for survival analysis was time to first catheter failure requiring catheter removal or exchange. Failure was strictly defined as catheter replacement or removal necessitated by: (1) dysfunction or occlusion, (2) fibrin sheath formation, (3) thrombosis, (4) infection, or (5) mechanical failure. Elective catheter removal following the successful maturation of an arteriovenous fistula was explicitly excluded from the definition of failure and was treated as a censoring event. Each of these endpoints required documentation by an interventional radiologist and was independently verified through procedural records prior to inclusion as a failure event. This unified definition of catheter failure was applied consistently across all analyses. Catheter dysfunction is defined as the inability to achieve adequate dialysis flow, typically when the flow is less than 300 mL/min despite standard interventions. Thrombosis is identified as either a clinically suspected or imaging-confirmed thrombotic occlusion. Fibrin sheath formation is diagnosed through a fluoroscopic contrast study or venography, which shows a reduction in flow related to the sheath. Mechanical failure refers to issues such as catheter kinking, malposition, fracture, or other forms of structural compromise. Elective catheter removal following successful arteriovenous fistula maturation was explicitly excluded from this definition and treated as a censoring event.

### 2.4. Follow-Up

Following catheter placement, all patients were enrolled in a structured outpatient hemodialysis program at our institution or an affiliated dialysis unit. Clinical follow-up was conducted through prospectively maintained procedural and nephrology records, supplemented by electronic medical record review. Patients underwent hemodialysis sessions three times weekly, during which catheter performance was assessed by dialysis nursing staff and recorded. Catheter blood flow rates and venous pressures were documented at each session. Patients with blood flow rates consistently below 300 mL/min despite repositioning maneuvers, or with venous pressures exceeding thresholds established by unit protocol, were referred for interventional radiology evaluation. All catheter-related events, interventions, and clinical outcomes were recorded prospectively in the departmental interventional radiology database and subsequently abstracted for this analysis. Patients were monitored from the time the catheter was placed until either a failure event occurred or they were censored. Follow-up data were collected from dialysis records and hospital information systems. Patients were censored in cases where they transitioned to permanent vascular access (such as an arteriovenous fistula), died without experiencing catheter failure, reached the end of the study follow-up period, or were lost to follow-up. Clinical assessments were routinely conducted during dialysis sessions, with additional imaging techniques (such as fluoroscopy or venography) performed when catheter dysfunction was suspected. Follow-up data were censored at a predefined data-lock date of December 2025 corresponding to the final date of data extraction from institutional records.

### 2.5. Data Collection

The following variables were recorded: age, sex, primary diagnosis, comorbidities (diabetes mellitus [DM], hypertension [HTN]), baseline laboratory values (hemoglobin, platelet count, INR), dialysis vintage (duration of prior hemodialysis in months), catheter location, catheter type, catheter length, and antiplatelet medication use. Catheter-related bloodstream infection (CRBSI) was diagnosed according to standard Infectious Diseases Society of America criteria.

### 2.6. Statistical Analysis

Statistical analysis was performed using SPSS Statistics version 25.0 (IBM Corp., Armonk, NY, USA) for descriptive statistics, and R software (version 4.2.1, R Foundation for Statistical Computing, Vienna, Austria) with the ‘coxphf’ and ‘survRM2’ packages for advanced survival modeling. Descriptive statistics were expressed as frequency and percentage for categorical variables and as mean ± standard deviation or median (interquartile range, IQR) for continuous variables.

No formal a priori sample size calculation was performed, as this was a retrospective observational study utilizing all eligible patients identified within the defined enrollment period. The resulting sample size of 189 patients, with 13 failure events in the non-heparin-coated group and zero events in the heparin-coated group, provided sufficient events to detect a statistically significant difference in Kaplan–Meier survival curves via the Log-Rank test. However, the limited number of total failure events constrained the statistical power of the multivariable Cox regression model, and the absence of events in the heparin-coated group necessitated the use of penalized estimation methods. These constraints are acknowledged as limitations of the present analysis, and the findings of the multivariable model should be interpreted accordingly. Catheter failure-free survival was analyzed using the Kaplan–Meier method. Patients were censored at the time of transition to definitive vascular access (arteriovenous fistula), death without prior catheter failure, end of available study follow-up, or loss to follow-up. Survival differences were evaluated using the Log-Rank test.

To evaluate predictors of catheter failure, a multivariable survival analysis was planned. Because zero failure events occurred in the heparin-coated group causing complete separation, standard partial likelihood estimation was undefined. Accordingly, Firth’s penalized partial likelihood Cox regression was applied to reduce small-sample bias and obtain finite point estimates [10]. Restricted mean survival time (RMST) was also calculated to describe survival differences since the median was not reached in the heparin group. Statistical significance was defined as *p* < 0.05.

## 3. Results

### 3.1. Patient Demographics and Baseline Characteristics

A total of 189 patients were included in the study. The mean age was 64.43 ± 16.11 years (median 67 years; range 19–93 years). The cohort comprised 100 male (52.9%) and 89 female (47.1%) patients. Comorbidities were present in 155 patients (82.0%), with hypertension identified in 107 (56.6%) and diabetes mellitus in 97 (51.3%). Antiplatelet therapy was used by 76 patients (40.2%). Baseline laboratory values showed a mean hemoglobin of 9.54 ± 1.59 g/dL, mean platelet count of 214.57 ± 81.30 × 10^3^/µL, and a mean INR of 1.18 ± 0.15. Dialysis vintage showed marked dispersion, with a median of 1.0 months (IQR: 1.0–24.0; range 1–180 months).

Detailed demographic and clinical characteristics are presented in Table 1. The most common catheter insertion site was the right internal jugular vein (148 patients; 78.3%). Heparin-coated catheters were used in 80 patients (42.3%), whereas non-heparin-coated catheters were placed in 109 patients (57.7%).

### 3.2. Comparison of Heparin-Coated and Non-Heparin-Coated Groups

Baseline demographic, laboratory, and procedural characteristics were broadly comparable between the heparin-coated and non-heparin-coated catheter groups, with no statistically significant differences observed in age, sex distribution, hemoglobin, platelet count, INR, dialysis vintage, prevalence of DM or HTN, antiplatelet use, or catheter insertion site (all *p* > 0.05; Table 2).

With respect to clinical outcomes (Table 3), the rate of thrombotic and mechanical complications was significantly higher in the non-heparin-coated group: 14 of 109 patients (12.8%) experienced a complication compared with 1 of 80 patients (1.3%) in the heparin-coated group (*p* = 0.004). Catheter exchange was required in 20 patients (18.3%) in the non-heparin-coated group versus none in the heparin-coated group (*p* < 0.001). During follow-up, clinical infection occurred in 10 patients (9.2%) in the non-heparin group and 6 patients (7.5%) in the heparin group (*p* = 0.683).

### 3.3. Kaplan–Meier Catheter Event-Free Survival Analysis

In the revised survival analysis, transition to arteriovenous fistula access was treated strictly as a censoring event. A catheter failure event occurred in 13 patients in the non-heparin-coated group and in 0 patients in the heparin-coated group. A total of 176 patients were censored during follow-up: 4 due to AV fistula creation, 17 due to death without prior catheter failure, and 155 reached the end of the study period or were lost to follow-up event-free. The difference in event occurrence was statistically significant (*p* < 0.001). In the non-heparin group, 13 unique patients experienced a qualifying failure event; these were utilized for the time-to-first-failure analysis. The total number of exchange procedures (*n* = 20) reported in Table 3 includes recurrent failures in these same patients after their initial event.

**Figure 2:**
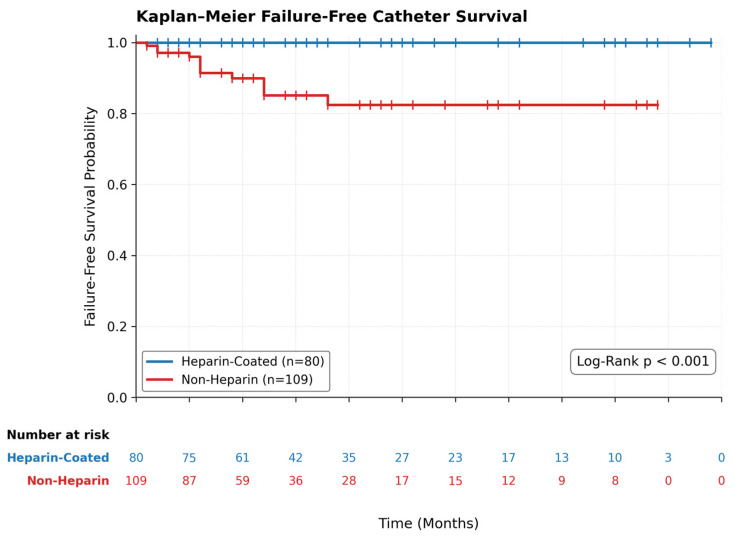
Kaplan–Meier catheter failure-free survival curves.

Restricted mean failure-free survival (RMST at τ = 49 months) was significantly longer in the heparin-coated group (49.0 months; the CI collapsed to zero variance as the survival function remained at 1.000 throughout follow-up) than in the non-heparin-coated group (41.7 months; 95% CI, 37.8–45.2). Kaplan–Meier analysis (Figure 2) confirmed significantly longer failure-free survival among heparin-coated catheter users (Log-Rank *p* < 0.001).

### 3.4. Predictors of Catheter Events

To identify independent predictors of catheter failure while accounting for follow-up time and censoring, multivariable Cox proportional hazards regression was performed (Table 4). Catheter type, age, INR, dialysis vintage, and hypertension were evaluated. Because no events occurred in the heparin-coated group, Firth’s penalized partial likelihood was used. The point estimate for heparin-coated catheters indicated a strong protective effect (HR = 0.087, 95% CI: 0.004–1.710, *p* = 0.108). Due to the limited overall number of failure events (*n* = 13), patient-level covariates including INR (*p* = 0.507), age (*p* = 0.743), dialysis vintage (*p* = 0.870), and hypertension (*p* = 0.295) did not independently predict failure. Adjustment for dialysis vintage did not materially change the HR for heparin-coated catheters.

## 4. Discussion

In this cohort, heparin-coated tunneled hemodialysis catheters were associated with significantly fewer complications and longer failure-free catheter survival than non-heparin-coated catheters. Specifically, complications occurred in only 1.3% of heparin-coated catheter recipients versus 12.8% in the non-heparin-coated group (*p* = 0.004). After arteriovenous fistula transition was treated as a censoring event, Kaplan–Meier analysis continued to show the same overall advantage. These findings are clinically relevant and are consistent with prior reports suggesting lower thrombotic catheter dysfunction with heparin-coated devices [8,11].

Previous studies have described several biologically plausible mechanisms through which heparin coating may reduce catheter-related complications. Heparin immobilized on catheter surfaces provides a sustained antithrombotic effect at the blood–material interface, inhibiting platelet adhesion, fibrin deposition, and downstream thrombus propagation [7,8]. Fibrin sheath formation is a clinically significant cause of tunneled catheter dysfunction; it encases the catheter tip, reduces blood flow, and serves as a nidus for biofilm formation and subsequent CRBSI [2,5]. Özdemir et al. [6] reported a comparable cohort of 161 patients, observing a significantly lower rate of fibrin sheath formation in the heparin-coated group (*p* = 0.017). Our data extend this evidence by demonstrating a significant Kaplan–Meier survival advantage using a rigorous time-to-event framework.

The complete absence of catheter exchange in the heparin-coated group is a noteworthy finding. This is consistent with the possibility that heparin coating reduces clinically relevant dysfunction, although the retrospective design precludes causal inference. While this result should be interpreted cautiously due to the retrospective design, it has potential implications for vascular access management [12].

In the multivariable penalized Cox regression analysis, patient-level covariates including INR, age, and dialysis vintage did not independently predict catheter failure. While factors like elevated INR and uremic coagulopathy are well-documented challenges in ESRD populations [13,14], and systemic conditions such as hypertension and anemia are known to drive endothelial injury and alter blood rheology [15,16], the lack of statistical significance in our model is primarily a function of the complete separation and low overall event rate (*n* = 13) rather than a definitive absence of biological relevance. This suggests that when a highly effective anti-thrombotic catheter coating is utilized, the device-level protection may partly offset these baseline physiological risks, contributing to the observed survival advantage, though larger multicenter cohorts are required to confirm this hypothesis.

Consistent with recent literature, the right internal jugular vein was the overwhelmingly preferred insertion site in our cohort (78.3%). Kanchanasuttirak et al. [17] demonstrated that catheter tip position at the pericavoatrial junction or right atrium is associated with optimal outcomes. Although our study did not systematically record catheter tip position on postoperative imaging, we observed no statistically significant differences in event rates between right and left IJV insertion sites.

Heparin-coated catheters generally have a higher initial procurement cost compared to standard polyurethane models. However, clinical results indicate that they may offer long-term economic benefits. By significantly extending the time before catheter failure and reducing the need for frequent exchanges, heparin-coated catheters could decrease the overall healthcare costs associated with managing end-stage renal disease (ESRD). Each catheter exchange involves not only the price of a new device but also costs related to using the interventional radiology suite, staff wages, and potential hospitalization. Additionally, these catheters help preserve the patient’s limited central venous access, avoiding the issue of “exhausted access.” This characteristic makes them a more sustainable option either as a temporary solution leading to the maturation of a permanent arteriovenous fistula (AVF) or as a long-term alternative for patients who are not suitable candidates for surgical access [4,6].

This study has several limitations that should be considered when interpreting its findings. First, the retrospective, single-center design introduces inherent selection bias, which limits the generalizability of the results to other institutions, patient populations, and catheter systems. Second, and perhaps most importantly, the allocation of catheter types was non-randomized and determined by operator preference and device availability at the time of the procedure. This introduces the potential for unmeasured confounding factors. However, it is worth noting that the baseline characteristics were statistically comparable between groups. It is impossible to exclude the influence of subtle, undocumented biases from operators in their device selection. The direction and magnitude of any such biases cannot be determined from the available data. Third, catheter length, which varied from 19 to 33 cm across the cohort, was not included as a variable in the multivariable model due to the limited number of failure events. This means that differences in catheter length between groups and any associated effects on tip position and hemodynamics remain uncontrolled variables. Fourth, catheter tip position was not systematically recorded in post-procedural imaging, preventing an evaluation of its contribution to outcomes. Fifth, dialysis adequacy data (Kt/V) were not available for this cohort. Catheter-related flow limitations may have influenced dialysis dosing in ways that were not captured by the primary failure endpoint. Finally, the absence of failure events in the heparin-coated group, while representing the study’s most significant clinical finding, also rendered the multivariable Cox model underpowered. This made it difficult to identify individual patient-level predictors of failure and necessitated the use of penalized estimation methods, the results of which should be interpreted with caution. Overall, these limitations highlight the need for a prospective, randomized, multicenter investigation to confirm and expand upon the current observations.

The findings of this study should be regarded as hypothesis-generating rather than definitive, and several directions for future investigation are clearly indicated. This study should compare heparin-coated catheters with other advanced surface modifications, such as silver-ion or antibiotic-impregnated surfaces, across various patient populations. Future research should also focus on subgroup analyses, especially among patients with a history of recurrent thrombosis or hypercoagulable states, to assess whether heparin coating offers superior benefits in high-risk groups. Additionally, incorporating a formal cost–utility analysis into future study protocols would provide clinicians and hospital administrators with essential data to support the integration of heparin-coated devices into standard vascular access practices.

## 5. Conclusions

In this retrospective single-center study, heparin-coated tunneled hemodialysis catheters were associated with significantly longer failure-free survival and lower complication rates compared to non-heparin-coated catheters. These findings suggest that heparin-coated devices may merit consideration in selected ESRD patients requiring long-term tunneled access. Due to the zero-event rate in the heparin group, the multivariable model was underpowered to identify independent patient-level predictors such as INR. Multicenter collaborative studies with larger sample sizes are needed to establish evidence-based risk stratification tools for hemodialysis catheter management.

## Figures and Tables

**Table 1:** Baseline demographic and clinical characteristics of the study population (*n* = 189).

Characteristic	Overall Cohort (*n* = 189)
**Age** **, years (mean ± SD)**	64.43 ± 16.11; median 67 (range 19–93)
**Sex, male (** ***n*** **, %)**	100 (52.9%)
**Sex, female (** ***n*** **, %)**	89 (47.1%)
**Diabetes mellitus (** ***n*** **, %)**	97 (51.3%)
**Hypertension (** ***n*** **, %)**	107 (56.6%)
**Antiplatelet therapy (** ***n*** **, %)**	76 (40.2%)
**Hemoglobin, g/dL (mean ± SD)**	9.54 ± 1.59
**Platelet count, ×10^3^/µL (mean ± SD)**	214.57 ± 81.30
**INR (mean ± SD)**	1.18 ± 0.15
**Dialysis vintage, months**	Median 1.0 (IQR 1.0–24.0); range 1–180
**Catheter insertion site: right internal jugular**	148 (78.3%)
**Catheter insertion site: left internal jugular**	38 (20.1%)
**Catheter insertion site: right femoral**	3 (1.6%)
**Catheter type: heparin-coated**	80 (42.3%)
**Catheter type: non-heparin-coated**	109 (57.7%)

**Table 2:** Baseline characteristics according to catheter type.

Variable	Non-Heparin (*n* = 109)	Heparin (*n* = 80)	*p*-Value
**Age, years**	64.6 ± 16.3	64.2 ± 15.9	0.838
**Male sex**	57 (52.3%)	43 (53.8%)	0.843
**Female sex**	52 (47.7%)	37 (46.3%)	
**Hemoglobin, g/dL**	9.55 ± 1.69	9.53 ± 1.45	0.911
**Platelet count, ×10^3^/µL**	217.83 ± 81.22	210.14 ± 81.72	0.279
**INR**	1.18 ± 0.15	1.17 ± 0.14	0.700
**Dialysis vintage, months**	1.0 (1.0–21.0)	1.0 (1.0–24.0)	0.460
**Diabetes mellitus**	52 (47.7%)	45 (56.3%)	0.246
**Hypertension**	61 (56.0%)	46 (57.5%)	0.833
**Antiplatelet use**	40 (36.7%)	36 (45.0%)	0.250
**Right internal jugular access**	85 (78.0%)	63 (78.8%)	0.317
**Left internal jugular access**	21 (19.3%)	17 (21.3%)	
**Right femoral access**	3 (2.8%)	0 (0%)	

**Table 3:** Clinical outcomes and censoring profile according to catheter type.

Variable	Non-Heparin (*n* = 109)	Heparin (*n* = 80)	*p*-Value
**Any thrombotic/mechanical complication**	14 (12.8%)	1 (1.3%)	0.004
**Clinical infection**	10 (9.2%)	6 (7.5%)	0.683
**Patients with at least one catheter failure (** ***n*** **, %)**	13 (11.9%)	0 (0%)	<0.001
**Total catheter exchange procedures performed (** ***n*** **)**	20 procedures	0 procedures	<0.001
**Censored: AV fistula creation**	4	0	
**Censored: death without prior failure**	9	8	
**Censored: end of follow-up**	83	71	
**Censored: loss to follow-up**	0	1	
**Median follow-up, months**	12.0	15.0	

**Table 4:** Multivariable Cox proportional hazards regression analysis of predictors of catheter failure.

Variable	HR	95% CI	*p*-Value
**Heparin-coated catheter (vs. non-heparin)**	0.087	0.004–1.710	0.108
**Age, years**	0.990	0.932–1.051	0.743
**INR**	0.115	0.000–68.30	0.507
**Dialysis vintage, months**	0.997	0.962–1.034	0.870
**Hypertension**	3.055	0.377–24.760	0.295

## Data Availability

The data presented in this study are available on reasonable request from the corresponding author.

## Abbreviations

The following abbreviations are used in this manuscript:

AV	Arteriovenous
AVF	Arteriovenous fistula
CI	Confidence interval
CKD	Chronic kidney disease
CRBSI	Catheter-related bloodstream infection
DM	Diabetes mellitus
ESRD	End-stage renal disease
HTN	Hypertension
IJV	Internal jugular vein
LR	Likelihood ratio
OR	Odds ratio
SD	Standard deviation
RRT	Renal replacement therapy
TDC	Tunneled dialysis catheter
